# Multi-trajectory group profiles of well-being and associated predictors among adults experiencing homelessness and mental illness: findings from the At Home/Chez Soi study, Toronto site

**DOI:** 10.1007/s00127-021-02093-x

**Published:** 2021-04-17

**Authors:** Cilia Mejia-Lancheros, James Lachaud, Tim Aubry, Kathryn Wiens, Patricia O’Campo, Vicky Stergiopoulos, Stephen W. Hwang

**Affiliations:** 1 MAP Centre for Urban Health Solutions, Li Ka Shing Knowledge Institute, St Michael’s Hospital, Unity Health Toronto, 30 Bonds Street, Toronto, ON M5B 1W8 Canada; 2 School of Psychology, University of Ottawa, Ottawa, ON Canada; 3 Dalla Lana School of Public Health, University of Toronto, Toronto, ON Canada; 4 Centre for Addiction and Mental Health, Toronto, ON Canada; 5 Department of Psychiatry, University of Toronto, Toronto, ON Canada; 6 Division of General Internal Medicine, Department of Medicine, University of Toronto, Toronto, ON Canada

**Keywords:** Homeless person, Metal illness, Housing stability, Quality of life, Well-being status

## Abstract

**Purpose:**

To conduct a multi-dimensional and time-patterned analysis to identify distinct well-being trajectory profiles over a 6-year follow-up period among adults experiencing homelessness and mental illness.

**Methods:**

Data from 543 participants of the At Home Chez Soi study’s Toronto site were examined over a 6-year follow-up period, including measures of quality of life, community functioning, housing stability, and substance use. Well-being trajectories were identified using Group-Based Trajectory Modelling. Multinomial regression was used to identify predictor variables that were associated with each well-being trajectory profile.

**Results:**

Four well-being profiles were identified: low well-being, moderate well-being, good well-being, and high well-being. Factors associated with a greater likelihood of following a better well-being profile included receiving Housing First, reporting female gender and non-white ethnicity, having post-secondary studies, and reporting a high resilience level. Concurrently, factors associated with a lower likelihood of better well-being profiles were having a history of chronic homelessness, experiences of discrimination in the healthcare setting, having comorbid mental disorders and a high level of symptom severity, and reporting a history of traumatic brain injury and childhood adversity.

**Conclusions:**

Individuals experiencing homelessness follow distinct well-being profiles associated with their socio-demographic characteristics, health status, trauma history, resilience capabilities, and access to housing and support services. This work can inform integrated housing and support services to enhance the well-being trajectories of individuals experiencing homelessness.

**Trial registration:**

At Home/Chez Soi trial was registered with ISRCTN, ISRCTN42520374, http://www.isrctn.com/ISRCTN42520374.

**Supplementary Information:**

The online version contains supplementary material available at 10.1007/s00127-021-02093-x.

## Introduction

Homelessness is a daunting issue affecting millions of people worldwide. In North America, there are over 552,500 people experiencing homelessness in a single night in the United States [1] and 35,000 in Canada, where over 235,000 people experience homelessness in a given year. In Europe, there are approximately 700,000 people sleeping rough or staying in temporary or emergency accommodations each night [2]. The ability to improve the living conditions, health, and overall well-being of individuals experiencing homelessness remains a crucial social and public health challenge.

An individual’s well-being status is influenced by multiple objective and subjective aspects of their life; therefore, there is not a universal definition or a single set of indicators to fully capture well-being [3–5]. Economic stability, safe residential spaces, material deprivation, and physical and cognitive functioning are objective examples of well-being [4, 6]. Psychological, self-perceived health, quality of life, spirituality, social relations, and life satisfaction are subjective aspects of well-being [3, 4]. Subjective and objective well-being related factors are frequently interweaved over an individual’s life course [6]. Yet, well-being is seldom studied as a long-term outcome in the general population or groups of people experiencing homelessness, severe mental illness and poverty.

Due to their complex economic, social, housing, and physical and mental health needs [7], people experiencing homelessness tend to have less positive subjective and objective well-being outcomes [8, 9]. Few studies that have assessed multidimensional aspects of homeless people’s well-being mainly used cross-sectional [9, 10] or qualitative study designs [11]. Quality of life, psychosocial distress and self-esteem are aspects that some authors have combined to construct an overall multi-dimensional well-being status [10]. Other authors have used specific well-being indexes, such as the World Health Organization-Five Well-being Index [9]. Aspects such as food, income, housing, health, friendships, family, romantic relations, physical appearance, and life satisfaction have also been used and analyzed separately to capture specific well-being domains (material, social, and satisfaction domains) well-being of homeless populations [9]. In qualitative studies, well-being among people experiencing homelessness has been explored on a domain-specific basis (e.g., physical, mental, and social well-being) rather than an overall construct [11].

No studies have assessed homeless people's well-being as a construct of multiple intertwined well-being-related aspects that closely evolved over time. Yet, the overall well-being of individuals experiencing homelessness could be a result of several interlaced circumstances including social factors (e.g., discrimination and stigma, lack of access to social and health services) and physical and mental illness, including alcohol and substance use disorders [12, 13]. Early-life trauma, social exclusion, and economic hardship are underlying risk factors for homelessness and mental illness [14–16] and contribute to the low well-being of those experiencing homelessness.

Housing state is a crucial determinant of well-being for homeless people [8, 17] and frequently intersect with other well-being determinants [18]. However, previous studies on homeless populations, have focused mainly on the impact of housing on key well-being-related aspects such as mental health symptoms, housing stability, functioning, and quality of life, assuming a sequential relationship. For instance, Housing First (HF) interventions, which promote housing without preconditions, and support mental health needs based on a consumer-choice approach [19], have consistently shown effectiveness in improving housing stability [20]. Nonetheless, HF has shown limited effects on other outcomes such as quality of life [21–23], substance use [20, 24], recovery [25, 26] and discrimination [27].

The At Home/Chez Soi (AH/CS) study was a large multi-site intervention of HF support (intensive case management (ICM) or assertive community treatment (ACT)) plus rent supplement for adults with serious mental illness and a history of chronic homelessness conducted in five Canadian cities (Toronto, Moncton, Montreal, Winnipeg, and Vancouver) followed for a 2-year period (October 1, 2009, and March 31, 2013) [28]. The Toronto site of the AH/CS [29], received further 4-year funding to extend the duration of the study and examine the long-term effectiveness of HF on primary (i.e., housing stability) and secondary outcomes (e.g. quality of life, community functioning, mental and substance use disorders symptomatology, and access to health services) [21].

The 6-year follow-up analyses showed sustained greater improvements in housing stability (an essential aspect of objective well-being) among HF compared to the control participants [participants that received treatment as usual (TAU)] [21]. However, improvements in other well-being-related aspects such as self-reported quality of life, community functioning, and substances used were less marked, showing no statistically significant differences between HF and TAU groups [21]. Analyzing these key indicators of people's well-being with lived experiences of homelessness as separate identities rather than as interlocked and dynamic aspects that could evolve are less informative to understand the complex relationships and the main contributing factors of well-being in this population.

Leveraging the long-term collected data on the primary and secondary outcomes of participants in the AH/CS Toronto site in this study, we used the group-based trajectory modelling (GBTM) approach [30, 31] to identify the specific longitudinal and multifaceted well-being profiles for individuals experiencing homelessness and mental illness. To construct the well-being profiles, we combined the six-year trajectories of the AH/CS Toronto primary outcome (housing stability) and secondary and exploratory outcomes (quality of life (QoLi), community functioning, and alcohol and substance use severity), which are key aspects of objective and subjective well-being, respectively. In addition, these outcomes are considered central benefits to receiving HF [32]. We also identified key factors (socioeconomic factors, physical and mental disorders, childhood trauma, and adult resilience) associated with the likelihood of following the identified well-being group profiles.

## Methods

### Study population and design

The present study population included Phase I and Phase II participants of the Toronto site of the AH/CS study [29]. The study’s Phase I, was part of the multi-site AH/CS study, a randomized trial on HF in 5 Canadian cities: Toronto, Winnipeg, Montreal, Moncton, and Vancouver [28]. It enrolled 575 participants during October 1, 2009, and March 31, 2013 [28]. To be included, participants needed to fulfill the following criteria. (1) Eighteen years old or over, (2) homeless or precariously housed (living in a rooming house, single-room occupancy, or a hotel or motel with two or more episodes of homelessness in the previous year, or being homeless for at least four weeks in the previous year), (3) serious mental or substance use disorder, assessed using the Mini-International Neuropsychiatric Interview (MINI) [33] and (4) legal status in Canada. Participants were stratified by mental health need level and randomized to receive the HF intervention [ICM or ACT plus rent supplements] or treatment as usual (TAU). TAU comprised access to the support services available in Toronto. To be classified as high needs, participants required a score of ≤ 62 on the Multnomah Community Ability Scale (MCAS) [34], psychotic or bipolar disorder, and either comorbid substance use, a recent mental health hospitalization, or incarceration [29].

In 2014, 2 years after completing the multi-site study, the Toronto-site received additional funding to extend the follow-up for up to 4 years more (January 2014–March 31, 2017) (Phase II). Thus, participants re-consented to continue their participation in an extended follow-up (Phase II). Of 575 participants, 414 agreed to their continuation in the study. The description of the Toronto AH/CS participants has been previously published [21].

For this study, 543 (94.4%) of the 575 participants were included if they had at least three-repeated measurements available either in Phase I or Phase II for all four indicators used for estimating the multidirectory well-being profiles. Except for the HF intervention group, there were no differences in the demographic, socioeconomic, and health profiles between the group of participants with complete data and those with incomplete data (Supplementary Table 1).

### Ethics approvals

The Toronto AH/CS study received ethics approval from the Research Ethics Board of St. Michael’s Hospital. Participants provided written informed consent to participate in the AH/CS study. The AH/CS study is registered with the International Standard Randomized Control Trial Number Register (ISRCTN42520374).

### Study measures

#### Main outcome

Well-being trajectory profiles were estimated using the following objective and subjective well-being measures [8, 17], which were assessed repeatedly as outcomes of the Toronto AH/CS study [21]. (1) *Housing stability* (objective well-being measure). It was assessed every three months over Phase I and every six months over Phase II of the Toronto AH/CS study using the Residential Time-Line Follow-Back (RTLFB) questionnaire [35]. The RTLFB captured the number of days participants spent living in different types of accommodations (e.g., their own housing unit, shelter, streets). In the present study, participants were considered to be stably housed if they spent at least 75% of accounted for days in stable housing accommodations (living in rental housing unit tenancy rights or expected to remain for six months or more in the same accommodation unit) at six months interval over Phases I and II. (2) *Self-reported Global QoLi* **(**subjective wellbeing measure). It was assessed every six months over Phases I and II using the 20-item Lehman’s 20-item QOL interview [36]. The overall QoLi score was derived from summing each item score in the 20-item QOL interview (rated on a 7-point scale). The overall score ranged from 20 to 140, with higher values indicating the greater quality of life. (3) *Community functioning* (subjective well-being-measure) was measured every 6 months over Phases I and II using the 17-item MCAS [34] to assess participants’ ability to function in the community in the areas of health, adaption, social skills, and behaviors. The overall score (range 17–85) was obtained by summing the scores for each of 17 items. Higher score values indicating higher community functioning. (4) *Substance use severity symptoms* (subjective wellbeing measure) were assessed every six months over Phases I and II using the overall score (range 0–5) of the five-item Global Appraisal of Individual Needs–Short Screener [37]. Higher values denote greater substance use severity in the past month.

#### Main predicting factors

Based on the previous well-being-related literature in homelessness [8–10] and considering the health, trauma, and resilience-related factors that homeless people frequently experienced [12, 13, 38, 39], we assessed the following factors as predictors of the identified multi-trajectory well-profiles.

*Housing first intervention*: receiving HF-treatment comprising ACT or ICM support services and rent supplements vs TAU.

*Socio-demographic factors:* age (years), gender (women/men) ethno-racial background (non-white/white), education (less than high school, completed high school, attended/completed graduate or postgraduate studies), lifetime duration of homelessness (< 3 years/ ≥ 3 years), and history of discrimination experiences in healthcare settings due to mental disorders (no/yes).

*Mental health problems:* number of mental disorders, excluding substance or alcohol use disorders (< 2/ ≥ 2), measured with the MINI screener [33]. Alcohol abuse or dependence disorder (no/yes) and substance abuse or dependence disorder (no/yes) also measured with the MINI. The severity of the mental illness symptoms was measured with the 14-item Colorado Symptom Index (score range: 14–70) [40, 41].

*Physical health problems:* number of chronic physical health conditions (CDs) (< 2/ ≥ 2) among asthma, chronic bronchitis or emphysema, tuberculosis, HIV/AIDS, migraine, stroke, heart disease, Alzheimer’s disease or dementia, dental problems, arthritis, an ulcer, Crohn’s disease or colitis, kidney or bladder problems, hypertension, a thyroid condition, diabetes, liver disease other than hepatitis, cancer, anemia [42, 43]. History of lifetime’s traumatic brain injury (TBI) (no/yes) [44].

*Early life trauma:* Adverse Childhood Experiences (ACEs) score (0–10) [45].

*Adulthood resilience* levels (range 0–8) measured with the Connor-Davidson Resilience Scale (CD-RISC2 scale) [46].

### Statistical analysis

The analysis was conducted in two steps. First, the identification of the multi-trajectory well-being profile groups. Second, the testing of associations between the well-being profile groups (outcome) and the main predictors previously described.

*Identification of the multi-trajectory well-being profile groups:* we identified the number and shape of the trajectories for each of the well-being measures (stable housing, QoLi, community functioning, and substance use severity) based on a combination of the cubic, quadratic, linear, and intercept polynomic using the statistical Group-Based Trajectory Modelling (GBTM) approach (finite mixture modelling) [30, 31]. We used the statistic Stata “*Traj”* program [47].

The number of trajectories and the shape of the trajectory groups was selected based on the Bayesian information criterion (BIC) (lower value indicates the best model). After the identification of the trajectory group for each of the well-being measures, we combined all well-being trajectory groups using the multi-trajectory statistical functions of the GBTM program (“*multgroups*”* and *“*multtrajplot*”) [30]. Again, different polynomic combinations (cubic, quadratic, linear, and intercept) of four-group based estimations were performed to identify the best multi-trajectory group profile model [30, 31].

We selected the best well-being multi-trajectory group model based on the best estimate for the BIC values (Supplementary Table 2) and the Group Average Posterior Probability (> 75%), weighted odds of correct classification (> 5.0), and the total posterior probabilities (Supplementary Table 3). The growth parameters estimations of the selected well-being Multi-trajectory group model are presented in Supplementary Table 4.

*Testing of the associations between the well-being profile groups and the main predictors:* we used multinomial regression models to analyze the association [Relative Risk Ratio (RR)] between the predicting factors and the well-being of multi-trajectory groups’ profiles. Some of the predicting factors have missing values data (ACES score = 18.2%, resilience score = 13.4%, CSI score = 3.7%, education = 3.5%, duration of homelessness = 5.5%, TBI = 4.2%, history of discrimination experiences within healthcare settings = 3.7%) (Supplementary Table 5). Thus, we imputed their missing values using Multiple Imputation (MI) via chained equations [48].

To increase the estimation precision and reduce the Monte Carlo error [49], we carried out 100 MI datasets. In the MI model, we include all the predicting factors and the well-being multi-trajectory groups’ profiles. The completed imputed values showed good appropriateness [50] (examples of imputed model appropriateness are in Supplementary Tables 6 to 10).

All the analyses were performed at a 95% confidence interval and tested at a 0.05 statistical significance level using Stata Software (version 16).

## Results

At baseline, our study population (*N* = 543) was on average 40.27 years old, men (68.32%), of non-white ethno-racial background (59.3%), experiencing lifetime chronic homelessness (54.39%), and receiving the HF intervention (53.8%).

### Well-being multi-trajectory group profiles

**Fig. 1:**
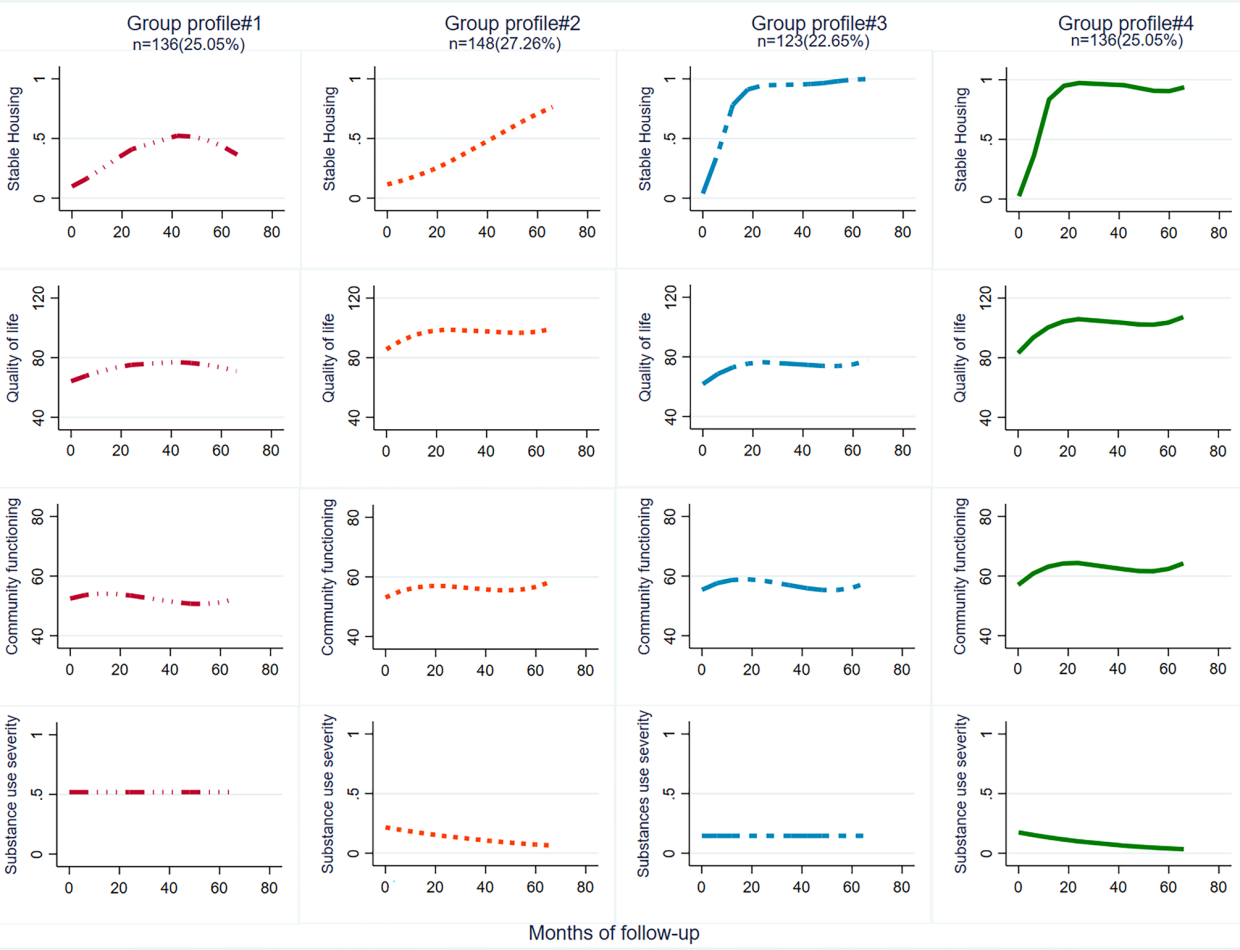
Participants’ well-being multitrajectory profiles over six-year follo-up, AH/CS study, Toronto site. Profile#1 = low well-being, Profile#2 = moderate well-being, Profile#3 = good well-being, Profile#4 = high well-being

Four well-being multi-trajectory profiles were identified based on housing stability, QoLi, community functioning, and substance use severity measures over 6 years (Fig. 1). Well-being *profile#1* (“low well-being”) represented 25.0% (*n* = 136) of study participants. The individuals in this group had low housing stability and QoLi, poor community functioning, and moderate to high substance use severity. Well-being *profile #2* (“moderate well-being”) comprised 27.3% (*n* = 148) of participants. These individuals experienced a moderate improvement in their housing stability, had a moderately high QoLi, moderate community functioning, and declining substance use severity. Well-being *profile#3* (‘good well-being”) included 22.7% (*n* = 123) of participants. These individuals had an immediate increase in stable housing status (sustained after 20 months), moderate QoLi, moderate levels of community functioning, and consistently low substance use severity. Well-being *profile #4* (“high well-being”) comprised 25.0% (*n* = 136) participants. This group demonstrated an immediate increase in housing stability, an early increase in QoLi and community functioning, and reduced substance use severity.

The BIC values (Supplementary Table 2) and the average posterior probability and the odds of correct classification (Supplementary Table 3) indicated a well-specification of the model. The specific estimates of model growth parameters are presented in Supplementary Table 4.

### Participants’ characteristic across the well-being multi-trajectory profiles

Table 1 describes the main baseline sample’s characteristics across their well-being profiles. T had the lowest percentage of HF treatment group participants (38%), women (25%), non-white ethnicity (41%), post-secondary studies (20%), and the highest percentage of participants with less than high school (62%), history of chronic homelessness (73%), at least two mental disorders (42%), alcohol abuse or dependence (65%), substance abuse or dependence (69%), at least two CDs (62%), and history of TBI (71%). In addition, these participants had the highest mean ACE score (5.3; SD = 2.7) and severity of mental illness symptoms (46.4; SD = 11.4), and the lowest mean resilience score (4.6; SD = 1.8). The *moderate well-being profile* had the lowest percentage of participants who experienced mental health discrimination in the healthcare setting (27%), had at least two mental disorders (18%), and had at least two CDs (50%). These participants also had the lowest severity of mental illness symptoms (34.6; SD = 13.3) and ACE score (3.2; SD = 2.8). The *good well-being profile* had the highest percentage of non-white ethnicity (68%), post-secondary education (41%), experiences of mental health discrimination (52%), and the lowest percentage of participants with less than high school (39%) and alcohol abuse or dependence (33%). The *high well-being profile* had the lowest percentage of participants with a history of chronic homelessness (39%), substance abuse or dependence (39%), history of TBI (44%), and the highest percentage of HF treatment group participants (73%) and women (43%). These participants had the highest mean resilience score (5.5; SD = 1.8).

**Table 1:** Participant baseline characteristics in the overall study sample and across well-being trajectory group profiles over six-years follow-up, AH/CS study, Toronto site (observed data)

Characteristics at baseline	*n*	Overall (*N* = 543)	Well-being multi-trajectory group profiles				*p* value
			Profile#1^a^ (*n* = 136)	Profile#2^b^ (*n* = 148)	Profile#3^c^ (*n* = 123)	Profile#4^d^ (*n* = 136)	
		%	%	%	%	%	
HF intervention group	**543**						
TAU	251	46.22	61.76	60.14	33.33	27.21	< 0.001^†^
HF Treatment (ACT/ICM)	292	53.78	38.24	39.86	66.67	72.79	< 0.001^‡^
Demographic factors							
Age (years)	**543**	40.27 (11.71)	39.87 (10.56)	40.29 (12.82)	42.06 (10.35)	39.03 (12.58)	0.207^§^
Gender	**543**						
Men	371	68.32	75.00	69.59	72.36	56.62	0.006
Women^e^	172	31.68	25.00	30.41	27.64	43.38	0.003
Ethno-racial background	**543**						
White	221	40.70	58.09	38.51	31.71	33.82	< 0.001
Non-white	322	59.30	41.91	61.49	68.29	66.18	< 0.001
Socioeconomic factors							
Education studies	**524**						
Less than high school studies	253	48.28	61.83	48.20	38.52	43.94	0.005
Completed high school studies	98	18.70	18.32	17.99	20.49	18.18	< 0.001
Attended/completed graduate or postgraduate studies	173	33.02	19.85	33.81	40.98	37.88	
Lifetime homelessness duration	**513**						
< 3 years	234	45.61	27.13	44.03	50.85	60.61	< 0.001
≥ 3 years	279	54.39	72.87	55.97	49.15	39.39	< 0.001
Discrimination experiences in health settings due to mental health problems	**523**						
No	316	60.42	51.91	73.38	48.36	66.41	< 0.001
Yes	207	39.58	48.09	26.62	51.64	33.59	0.333
Mental health problems							
Number of mental disorders (excluded substance use or alcohol disorders)^f^	**543**						
< 2	310	57.09	58.09	81.76	60.98	59.56	0.041
≥ 2	233	42.91	41.91	18.24	39.02	40.44	0.341
Alcohol abuse or dependence disorder	**543**						
No	310	57.09	35.29	62.16	66.67	64.71	< 0.001
Yes	233	42.91	64.71	37.84	33.33	35.29	< 0.001
Substance abuse or dependence disorder	**543**						
No	288	53.04	30.88	60.81	59.35	61.03	< 0.001
Yes	255	46.96	69.12	39.19	40.65	38.97	< 0.001
Severity of the mental illness symptoms							
CSI score (range 14–70)	**523**	40.23 (12.90)	46.42 (11.38)	34.61 (13.31)	43.31 (11.08)	37.10 (12.04)	< 0.001
Physical health problems							
Number of chronic diseases (CDs)^g^	**543**						
< 2 CDs	236	43.46	38.24	50.00	39.02	45.59	0.150
≥ 2 CDs	307	56.54	61.76	50.00	60.98	54.41	0.565
History of lifetime’s traumatic brain injury (TBI)	**520**						
No	239	45.96	29.23	53.28	44.63	56.06	< 0.001
Yes	281	54.04	70.77	46.72	55.37	43.94	< 0.001
Early life trauma							
ACEs score (range 0–10)	**444**	4.10 (2.86)	5.33 (2.71)	3.19 (2.77)	4.43 (2.7)	3.68 (2.74)	< 0.001
Adulthood resilience level							
Resilience score (CD-RISC2 scale) (range 0–8)	**470**	5.01 (1.99)	4.63 (1.83)	5.01 (2.05)	4.81 (2.18)	5.53 (1.78)	0.0035

Bold value indicates the number of participants with valid observed data

^a^Well-being profile #1 (“low well-being”)

^b^Well-being profile #2 (“moderate well-being”)

^c^Well-being profile #3 (“good well-being”)

^d^Well-being profile #4 (“high well-being”)

^e^Female category includes nine transsexual or transgender participants

^f^Major depressive episode, manic episode or hypomanic episode, PTSD, panic disorder, current mood disorder with psychotic features, current psychotic disorder

^g^Asthma, chronic bronchitis or emphysema, tuberculosis, HIV/AIDS, migraine, stroke, heart disease, Alzheimer’s disease or dementia, dental problems, arthritis, an ulcer, Crohn’s disease or colitis, kidney or bladder problems, hypertension, a thyroid condition, diabetes, liver disease other than hepatitis, cancer, anemia.

† = Chi-squared text. ‡ = *p *trend test. § = ANOVA *F *test.

### Potential predicting factors and well-being multi-trajectory profiles

The unadjusted and adjusted associations (RR) between baseline participants’ characteristics and the probability of following each multi-trajectory well-being profile are presented in Tables 2, 3, 4 and 5. *The low well-being profile* was the reference group for all models.

**Table 2:** Unadjusted and adjusted associations (Relative-risk ratio: RR) of Housing First treatment and demographic factors with well-being multi-trajectory group profiles for AH/CS study participants over 6-years follow-up, Toronto site (imputed data)

(*N* = 543)	Well-being multi-trajectory group profiles					
	Profile#2^b^ (*n* = 148) vs profile#1^a^		Profile#3^c^ (*n* = 123) vs profile#1^a^		Profile#4^d^ (*n* = 136) vs profile#1^a^	
	RR (95% CI)	*p* value	RR (95% CI)	*p *value	RR (95% CI)	*p* value
Housing First treatment and demographic factors						
Unadjusted association						
HF-treatment vs TAU	1.07 (0.66–1.73)	0.779	3.23 (1.94–5.38)	< 0.001	4.32 (2.59–7.21)	< 0.001
Age (years)	1.00 (0.98–1.02)	0.758	1.02 (1.00–1.04)	0.133	0.99 (0.97–1.01)	0.552
Women vs men	1.31 (0.78–2.21)	0.310	1.15 (0.66–1.99)	0.629	2.30 (1.37–3.85)	0.002
Non-white vs white ethno-racial background	2.21 (1.38–3.56)	0.001	2.99 (1.79–4.97)	< 0.001	2.71 (1.66–4.44)	< 0.001
Adjusted association^e^						
HF treatment vs TAU	1.10 (0.68–1.79)	0.705	3.49 (2.06–5.92)	< 0.001	4.43 (2.61–7.51)	< 0.001
Age (years)	1.01 (0.99–1.03)	0.300	1.03 (1.01–1.06)	0.007	1.01 (0.99–1.03)	0.335
Women vs men	1.30 (0.77–2.22)	0.328	1.16 (0.65–2.07)	0.617	2.25 (1.31–3.87)	0.003
Non-white vs white ethno-racial background	2.31 (1.42–3.76)	0.001	3.43 (2.01–5.86)	< 0.001	2.89 (1.71–4.88)	< 0.001

^a^Well-being profile#1 (“low well-being”)

^b^Well-being profile #2 (“moderate well-being”)

^c^Well-being profile#3 (“good well-being”)

^d^Well-being profile #4 (“high well-being”)

^e^Mutually adjusted model

**Table 3:** Unadjusted and adjusted associations (RR) of socioeconomic factors and discrimination experiences with well-being multi-trajectory group profiles for AH/CS study over six-years follow-up, Toronto site (imputed data)

(*N* = 543)	Well-being’s multi-trajectory group profile					
	Profile#2^b^ (*n* = 148) vs profile#1^a^		Profile#3^c^ (*n* = 123) vs profile#1^a^		Profile#4^d^ (*n* = 136) vs profile#1^a^	
	RR (95% CI)	*p *value	RR (95% CI)	*p *value	RR (95% CI)	*p *value
Socioeconomic factors						
Education						
Unadjusted association						
Completed high school studies vs less than high school studies	1.27 (0.66–2.43)	0.475	1.80 (0.93–3.50)	0.083	1.41 (0.73–2.72)	0.309
Attended/completed graduate or postgraduate studies	2.18 (1.22–3.88)	0.008	3.26 (1.80–5.91)	< 0.001	2.63 (1.47–4.71)	0.001
Adjusted association^e^						
Completed high school studies vs less than high school studies	1.18 (0.61–2.29)	0.621	1.83 (0.91–3.69)	0.092	1.55 (0.76–3.13)	0.226
Attended/completed graduate or postgraduate studies	2.07 (1.14–3.74)	0.016	3.11 (1.66–5.84)	< 0.001	2.71 (1.45–5.07)	0.002
Lifetime homelessness duration						
Unadjusted association						
≥ 3 year vs < 3 years	0.46 (0.28–0.77)	0.003	0.36 (0.21–0.61)	< 0.001	0.24 (0.14–0.40)	< 0.001
Adjusted association^e^						
≥ 3 years vs < 3 years	0.48 (0.28–0.82)	0.008	0.35 (0.19–0.61)	< 0.001	0.25 (0.14–0.43)	< 0.001
Discrimination experiences in health settings due to mental health problems						
Unadjusted association						
Yes vs no	0.39 (0.23–0.64)	< 0.001	1.13 (0.69–1.85)	0.629	0.48 (0.28–0.82)	0.008
Adjusted association^e^						
Yes vs no	0.43 (0.26–0.72)	0.002	1.49 (0.88–2.53)	0.139	0.64 (0.37–1.09)	0.098

^a^Well-being profile #1 (“low well-being”)

^b^Well-being profile #2 (“moderate well-being”)

^c^Well-being profile #3 ("good well-being”)

^d^Well-being profile #4 (“high well-being”)

^e^Adjusted for HF intervention group, age, gender, and ethno-racial background.

**Table 4:** Unadjusted and adjusted associations (RR) of mental health and physical health factors with well-being multi-trajectory group profiles for AH/CS study over six-years follow-up, Toronto site (imputed data)

(*N* = 543)	Well-being’s multi-trajectory group profile					
	Profile#2^b^ (*n* = 148) vs profile#1^a^		Profile#3^c^ (*n* = 123) vs profile#1^a^		Profile#4^d^ (*n* = 136) vs profile#1^a^	
	RR (95% CI)	*p* value	RR (95% CI)	*p* value	RR (95% CI)	*p* value
Mental health factors						
Number of mental disorders (excluding substance use or alcohol disorders)						
Unadjusted association						
≥ 2 mental disorders vs < 2	0.31 (0.18–0.53)	< 0.001	0.89 (0.54–1.46)	0.637	0.94 (0.58–1.53)	0.805
Adjusted association^e^						
≥ 2 mental disorders vs < 2	0.33 (0.19–0.57)	< 0.001	1.01 (0.59–1.70)	0.983	1.01 (0.60–1.70)	0.962
Alcohol abuse or dependence disorder						
Unadjusted association						
Yes vs no	0.33 (0.19–0.57)	< 0.001	0.27 (0.16–0.46)	< 0.001	0.30 (0.18–0.49)	< 0.001
Adjusted association^e^						
Yes vs no	0.37 (0.23–0.61)	< 0.001	0.31 (0.18–0.53)	< 0.001	0.36 (0.21–0.62)	< 0.001
Substance abuse or dependence disorder						
Unadjusted association						
Yes vs no	0.29 (0.18–0.47)	< 0.001	0.31 (0.18–0.51)	< 0.001	0.29 (0.17–0.47)	< 0.001
Adjusted association^e^						
Yes vs no	0.32 (0.19–0.54)	< 0.001	0.36 (0.21–0.63)	< 0.001	0.32 (0.18–0.55)	< 0.001
Severity of the mental illness symptoms						
Unadjusted association						
CSI score (range 14–70)	0.92 (0.90–0.94)	< 0.001	0.98 (0.96–0.99)	0.036	0.94 (0.92–0.96)	< 0.001
Adjusted association^e^						
CSI score (range 14–70)	0.92 (0.90–0.94)	< 0.001	0.98 (0.96–1.01)	0.109	0.94 (0.92–0.96)	< 0.001
Physical health factors						
Number of chronic diseases (CDs)						
Unadjusted association						
≥ 2 CDs vs < 2	0.62 (0.39–0.99)	0.047	0.97 (0.59–1.60)	0.896	0.74 (0.46–1.20)	0.220
Adjusted association^e^						
≥ 2 CDs vs < 2	0.66 (0.41–1.08)	0.099	1.05 (0.62–1.80)	0.846	0.85 (0.50–1.42)	0.529
History of lifetime traumatic brain injury						
Unadjusted association						
Yes vs no	0.37 (0.22–0.61)	< 0.001	0.51 (0.30–0.85)	0.010	0.33 (0.20–0.54)	< 0.001
Adjusted association						
Yes vs no	0.43 (0.25–0.72)	0.001	0.67 (0.38–1.18)	0.166	0.44 (0.25–0.76)	0.003

^a^Well-being profile#1 (“low well-being”)

^b^Well-being profile #2 (“moderate well-being”)

^c^Well-being profile#3 (“good well-being”)

^d^Well-being profile #4 (“high well-being”)

^e^Adjusted for HF intervention group, age, gender, and ethno-racial background.

**Table 5:** Unadjusted and adjusted associations (RR) of early childhood trauma and adulthood resilience with well-being multi-trajectory group profiles for AH/CS study over six-years follow-up, Toronto site (imputed data)

(*N* = 543)	Well-being’s multi-trajectory group profile					
	Profile#2^b^ (*n* = 148) vs profile#1^a^		Profile#3^c^ (*n* = 123) vs profile#1^a^		Profile#4^d^ (*n* = 136) vs profile#1^a^	
	RR (95% CI)	*p* value	RR (95% CI)	*p* value	RR (95% CI)	*p* value
Early life trauma						
Unadjusted association						
ACES score (range 0–10)	0.75 (0.68–0.83)	< 0.001	0.89 (0.81–0.98)	0.017	0.82 (0.75–0.90)	< 0.001
Adjusted association^e^						
ACES score (range 0–10)	0.75 (0.67–0.83)	< 0.001	0.91 (0.82–1.02)	0.094	0.81 (0.73–0.90)	< 0.001
Resilience level at study baseline						
Unadjusted association						
Resilience score (CD-RISC2 scale) (range 0–8)	1.10 (0.97–1.25)	0.133	1.03 (0.91–1.18)	0.627	1.24 (1.09–1.41)	0.001
Adjusted association^e^						
Resilience score (CD-RISC2 scale) (range 0–8)	1.11 (0.97–1.26)	0.120	1.04 (0.91–1.19)	0.569	1.27 (1.10–1.46)	0.001

^a^Well-being profile#1 (“low well-being”)

^b^Well-being profile #2 (“moderate well-being”)

^c^Well-being profile#3 (“good well-being”)

^d^Well-being profile #4 (“high well-being”)

^e^Adjusted for HF intervention group, age, gender, and ethno-racial background.

### Housing first treatment, demographic factors and well-being profiles

Table 2 reports the associations of the HF intervention and demographic factors with the probability of membership in each well-being profile**.** Participants in the HF treatment group were more likely to follow either the *good well-being* (RR: 3.49; 2.06–5.92) or *high well-being *(RR: 4.43; 2.61–7.51) profiles. Women were more likely to follow the *high well-being profile *(RR: 2.25; 1.31–3.87), while participants with a non-white ethno-racial background were likely to be in the *moderate well-being* (RR: 2.31; 1.42–3.76), *good well-being* (RR: 3.43; 2.01–5.86), or *high well-being profiles* (RR = 2.89; 1.71–4.88).

### Socioeconomic factors, discrimination experiences and well-being profiles

Table 3 reports the associations between socioeconomic factors and discrimination experiences and the probability of following each well-being profile. Following adjustment for HF intervention and demographic factors, people with post-secondary studies had a higher probability of following a positive well-being profile, including the *moderate well-being* (RR: 2.07; 1.14–3.74), *good-well-being* (RR: 3.11; 1.66–5.84), and *high well-being* (RR: 2.63, 1.47–4.71) profiles. History of chronic homelessness was associated with a lower likelihood of having a positive well-being profile, including *moderate well-being* (RR: 0.48; 0.28–0.82), *good well-being* (RR: 0.35; 0.19–0.61) and *high well-being* (RR: 0.25, 0.14–0.43). History of discrimination experienced in healthcare settings due to mental illness was associated with a lower probability of following the *moderate well-being* profile (RR = 0.39, 0.23–0.64).

### Mental and physical health factors and well-being profiles

Table 4 shows the associations between mental and physical health factors and the probability of following specific well-being profiles. Adjusting for HF intervention and demographic characteristics, a higher number of mental comorbidities was significantly associated with a lower likelihood of achieving the *moderate well-being profile* (RR: 0.33; 0.19–0.57). Participants with alcohol and substance use disorders were less likely to have a positive well-being profile, including moderate well-being, good well-being, and high well-being*.* Participants with higher mental illness symptoms severity were also less likely to have a *high well-being profile* (RR: 0.94; 0.92–0.96). History of TBI was significantly associated with a lower likelihood of following a *moderate well-being profile* (RR: 0.43; 0.25–0.72) or high well-being profile (RR: 0.44; 0.25–0.76).

### Early life trauma, adulthood resilience and well-being profiles

Table 5 reports the associations of childhood trauma and resilience with the probability of following each well-being trajectory. After adjusting for HF intervention and demographic factors, participants with a higher number of ACEs were less likely to attain any positive well-being profile, specifically the *moderate well-being* (RR: 0.75; 0.67–0.83) and *high well-being* (RR: 0.81; 0.73–0.90) profiles. Participants with higher resilience values also had a higher probability of following the *high well-being profile* (RR: 1.27; 1.10–1.46).

## Discussion

To the best of our knowledge, this is the first study to assess the longitudinal well-being profiles of people experiencing homelessness and mental health problems using a multi-dimensional and time-patterned analysis methodology [30, 51]. We identified four heterogeneous well-being profiles based on housing stability, QoLi, community functioning ability, and substance use severity over a 6-year period. These findings also identified several demographic, socioeconomic, childhood exposure, and health predictors of the identified well-being trajectory group profiles.

In our study, Housing First (rent-supplements plus mental health support services) was a strong predictor of having positive well-being multi-trajectory profiles. These findings add to the existing evidence of the effectiveness of HF for outcomes such as housing stability [20, 21], and its differential patterns or pathways [52]. Yet, previous studies have shown no conclusive results favoring HF over usual treatment regarding substance use, QoLi and community functioning ability [20, 21]. This may be explained by the complex and intertwined relationships of these key well-being measures within an individual, as well as heterogeneity between participants that were able to be captured by our examination of trajectories over time. Thus, our findings suggest that HF interventions indeed improve the multi-trajectories well-being profiles for subgroups of homeless people with severe mental health problems.

Women and non-white participants also achieved better well-being trajectories, which supports the presence of gender and ethno-racial based differences regarding social and health outcomes among homeless people [53, 54]. For example, homeless women have shown higher resilience and capability to cope with challenges compared to men [55] and have reported more life goals [56]. Yet, they tend to have more physical health problems and associated symptoms burden [57, 58] and mental health comorbidities [58, 59] than homeless men. Homeless men have been found to have a lower probability of reaching housing stability [60] and a higher probability of having alcohol and substance use problems than homeless women [61]. Regarding ethno-racial differences, higher levels of mental distress have been found among white homeless women than Black or Hispanic homeless women [62]. Finally, white homeless people have higher levels of substance use problems in men and show a higher likelihood of having mental disorders in women compared to non-white homeless people [63].

Regarding the predictive relationships of socioeconomic factors, participants with a higher level of education had a higher probability of achieving better well-being profiles. The converse relationship was observed for participants who had experienced more years of homelessness over the course of their lifetime. Education skills not only allow individuals to read, write and interpret text/documents and act as socioeconomic position indicators, but it is also an important determinant of life functioning and health [64] by allowing an individual to navigate across the complex structure of social systems and power, and make informed life and health decisions [65]. Previous studies have shown that higher literacy skills among homeless people help them gain more social and health benefits as such skills enable them to access resources and fulfill they everyday needs (identify food, clothing, sanitation, shelter options, accomplish health-related appointment or treatments) [65]. Education is also a determinant of health and social equity [66]; therefore, lack of education can contribute to, or perpetuate homelessness, poor well-being, and the associated driving and enhancing factors. In regards to chronic homelessness, studies report that people who spent more time homeless have worse mental health and poorer functioning [67, 68], and are less likely to achieve stable housing [52].

Mental illness severity and substance use disorders are important impeding factors in achieving better well-being profiles, as supported by past literature. For example, people with substance use disorders are less likely to gain stable housing [60], exit homelessness [69], or achieve better recovery trajectories [25]. People experiencing homeless frequently have a high prevalence of mental and substance use disorders [13], which often cluster with other poor health, behavioral, and social characteristics (e.g., poor physical health, victimization, adherence to the rehabilitation programs, discrimination/stigma, and criminal activity involvement) [27, 70–72]. Thus, these associated and intertwined adverse consequences of mental health and substance use disorders diminish further the possibility of positive well-being trajectories for this population group. Among other health-related factors, we found that participants with TBI history were less likely to follow more positive well-being trajectories. This shows that long-term impairment from head trauma can impact the social, health, and housing welfare of homeless people [44, 73].

Furthermore, we found that adverse events during childhood decreased the likelihood of having positive well-being trajectories in our homeless adult population. Studies have shown that ACES have detrimental long-term effects on health and social outcomes over the life course [74–76]. In a previous study of the same group of homeless adults, ACEs were strongly associated with higher psychopathology disorders [77]. Finally, we found that high levels of resilience were associated with a higher probability of attaining more positive well-being profiles. This suggests that there is a subgroup of homeless people that are able to adapt or leverage coping and resilience strategies [78, 79], which might help them overcome the challenges associated with being homeless and socially excluded.

The present study has some limitations. Our study participants were part of a pragmatic randomized trial of Housing First in a well-resourced setting such that the TAU group had access to numerous services and resources related to housing and mental health supports. Having a serious mental disorder was part of the inclusion criteria. Thus, findings may not be generalizable to other homeless population groups with dissimilar health profiles and residing in settings with less resources and services. As well-being is a multi-dimensional and subjective phenomenon, it is possible that other life aspects, not considered in the present study, could give different insights into well-being trajectories of this population. Yet, we used four key measures of well-being to capture the multi-dimensional well-being profiles of our study population including housing stability and community functioning assessed by interviewers. As well, we examined the effect of several predictive factors on the identified well-being trajectories.

Study findings have the following main implications for practice and policy. Individuals who experience homelessness are a heterogeneous group with specific life circumstances, needs, strengths, and abilities, which can improve or diminish their possibility to achieve better housing, health, and social outcomes and, therefore, shape their well-being trajectories. Thus, it is crucial to assess these individual differences when caring for and providing social and health support services to this population. Our study findings also highlight the need to consider the interactive relationships between health, housing, and social needs of individuals experiencing homelessness when planning, providing, and evaluating interventions and support services.

Considering a more comprehensive and multi-dimensional approach could help support this population enhance their possibility of successfully exiting the homeless and achieving better well-being profiles. This implies the provision of integrated services that combine housing supports with services tailored to an individual’s needs. Such services include: (1) access to mental health care, substance use treatment supports, and trauma-informed reduction services; (2) availability of spaces for engagement in meaningful social activities (e.g., expanding life skills such as cooking, using new technologies, work skills); (3) opportunities to build healthy networking and resources for developing resilience, and (4) facilitate reintegration and participation in their local communities and spaces through work, education, leisure, sports, and volunteer-related activities.

In conclusion, people experiencing homelessness and mental illness follow distinct well-being trajectory profiles based on their socioeconomic, health, childhood trauma, resilience-, and type of social support received. Comprehensive and multi-dimensional integrated support services and interventions could better enhance their social, health and housing outcomes.

## Supplementary Information

Below is the link to the electronic supplementary material.

Supplementary file1 (DOCX 36 KB)

## Data Availability

The At Home/Chez Soi study dataset cannot be made publicly available due to the sensitive nature of the data and agreements and procedures governing the use of the dataset established by the study sponsor, the Mental Health Commission of Canada. However, anonymized participant data from the AH/CS study, as well as the specific dataset used in the present paper, can be made available to investigators who complete the following steps: (1) present a study proposal that has received approval from an independent research committee or research ethics board; (2) provide a data request for review by the AH/CS data access committee; (3) following approval of the request, execute a data-sharing agreement between the investigators and the AH/CS data custodians. Study proposals and data access requests should be sent to Evie Gogosis (Evie.Gogosis@unityhealth.to), the research manager for the Toronto site of the AH/CS study, and Dr. Stephen Hwang (Stephen.Hwang@unityhealth.to), co-principal investigator of the Toronto site of the AH/CS study.

## Code availability

The statistical code used in this study could be accessed by contacting the corresponding author. The request will be reviewed by the AH/CS research team before it can be made available.
