# Supplementary material for: Multi-trajectory group profiles of well-being and associated predictors among adults experiencing homelessness and mental illness: findings from the At Home/Chez Soi study, Toronto site
Source: Soc Psychiatry Psychiatr Epidemiol. 2021 Apr 17;57(1):67–81. doi: 10.1007/s00127-021-02093-x (PMC8761129; doi:10.1007/s00127-021-02093-x)
Supplement: Supplementary file 1 — Supplementary file1 (DOCX 36 KB) [file 127_2021_2093_MOESM1_ESM.docx]

**Title: Multi-trajectory group profiles of well-being and associated predictors among adults experiencing homelessness and mental illness: findings from the At Home/Chez Soi study, Toronto site.**

**Supplementary Table 1. Comparison of the main baseline characteristics across the groups of participants with complete and incomplete data for the well-being profiles’ measures, AH/CS participants, Toronto.**

|  | **Complete data for the well-being measures**  **(housing stability, quality of life, community functioning and substance use severity)** | | | |
| --- | --- | --- | --- | --- |
| **Characteristics** |  | **Complete (n=543)** | **Incomplete (n=32)** |  |
| AH/CS study design variables | **n** | **% *or* mean (SD)** | **% *or* mean (SD)** | **p-value** |
| **HF intervention group** | **575** |  |  |  |
| TAU | 274 | 46.22 | 71.88 | 0.005 |
| HF Treatment | 301 | 53.78 | 28.13 |  |
| **Level of need for mental health services** | **575** |  |  |  |
| Moderate needs | 378 | 65.93 | 62.5 | 0.691 |
| High needs | 197 | 34.07 | 37.5 |  |
| **Demographic factors** |  |  |  |  |
| Age(years) | 575 | 40.27(0.50) | 41.19(2.36) | 0.667 |
| Gender | **575** |  |  |  |
| Men | 394 | 68.32 | 71.88 | 0.674 |
| Women | 181 | 31.68 | 28.13 | 0.669 |
| Ethno-racial background | **575** |  |  |  |
| White | 237 | 40.7 | 50 | 0.299 |
| Non-white | 338 | 59.3 | 50 |  |
| **Socioeconomic factors** |  |  |  |  |
| Education studies | **550** |  |  |  |
| Less than high school studies | 262 | 48.28 | 34.62 | 0.394 |
| Completed high school studies | 104 | 18.70 | 23.08 |  |
| Attended/completed graduate or  postgraduate studies | 184 |  |  |  |
| Lifetime homelessness duration | **538** |  |  |  |
| <3 years | 250 | 45.61 | 64.00 | 0.072 |
| ≥ 3 year | 288 | 54.39 | 36.00 |  |
| **Mental health problems** | **575** |  |  |  |
| Number of mental disorders (excluded substance use or alcohol disorders |  |  |  |  |
| <2 | 374 | 65.56 | 56.25 | 0.283 |
| ≥ 2 | 201 | 34.44 | 43.75 |  |
| Alcohol abuse or dependence disorder | **575** |  |  |  |
| No | 331 | 57.09 | 65.63 | 0.342 |
| Yes | 244 | 42.91 | 34.38 |  |
| Substance abuse or dependence disorder | **575** |  |  |  |
| No | 307 | 53.04 | 59.38 | 0.485 |
| Yes | 268 | 46.96 | 40.63 |  |
| **Physical health problems** |  |  |  |  |
| Number of chronic diseases (CDs) | **575** |  |  |  |
| <2 CDs | 251 | 43.46 | 46.88 | 0.705 |
| ≥ 2 CDs | 324 | 56.54 | 53.13 |  |

**Supplementary Table 2. BIC values for the different tested well-being multi-trajectories groups’ profiles models in the AH/CS participants (N=543) of the Toronto site.**

|  | **Multi-trajectory model: well-being groups profiles** | | | |
| --- | --- | --- | --- | --- |
| **Number of groups** | **Multi-trajectory models** | **Trajectory shapes** | **BIC**  (Number of observations: 16646) | **BIC**  (Number of participants: 543) |
|  | **Model 1** |  |  |  |
| 4 | Housing stability | 2 3 3 1 | -34872.40 | -34779.98 |
|  | Quality of life | 2 3 3 3 |  |  |
|  | Community functioning ability | 2 3 3 3 |  |  |
|  | Substance use severity | 0 0 1 1 |  |  |
|  | **Model 2** |  |  |  |
| 4 | Housing stability | 1 3 3 2 | -34834.85 | -34739.01 |
|  | Quality of life | 3 3 3 3 |  |  |
|  | Community functioning ability | 3 3 3 3 |  |  |
|  | Substance use severity | 1 0 0 1 |  |  |
|  | **Model 3** |  |  |  |
| 4 | Housing stability | 3 3 3 1 | -34797.23 | -34704.81 |
|  | Quality of life | 2 3 3 2 |  |  |
|  | Community functioning ability | 2 2 3 3 |  |  |
|  | Substance use severity | 1 1 1 0 |  |  |
| 4 | **Model 4** |  |  |  |
|  | Housing stability | 2 3 3 1 | -34786.29 | -34692.16 |
|  | Quality of life | 2 3 3 3 |  |  |
|  | Community functioning ability | 2 3 3 3 |  |  |
|  | Substance use severity | 1 1 1 0 |  |  |
|  | **Model 5** |  |  |  |
| 4 | Housing stability | 2 3 3 1 | -34877.90 | -34785.48 |
|  | Quality of life | 2 3 3 3 |  |  |
|  | Community functioning ability | 2 3 3 3 |  |  |
|  | Substance use severity | 1 0 0 1 |  |  |
|  | **Model 6** |  |  |  |
| 4 | Housing stability | 2 3 3 1 | -34793.58 | -34702.87 |
|  | Quality of life | 2 3 3 3 |  |  |
|  | Community functioning ability | 2 2 3 3 |  |  |
|  | Substance use severity | 0 0 1 1 |  |  |
|  | **Model 7^a^** |  |  |  |
| 4 | Housing stability | **2 3 3 1** | **-34770.89** | **-34676.76** |
|  | Quality of life | **2 3 3 3** |  |  |
|  | Community functioning ability | **3 3 3 3** |  |  |
|  | Substance use severity | **0 0 1 1** |  |  |
|  | **Model 8** |  |  |  |
| 4 | Housing stability | 2 3 3 1 | -34875.66 | -34784.95 |
|  | Quality of life | 2 3 3 2 |  |  |
|  | Community functioning ability | 2 3 3 3 |  |  |
|  | Substance use severity | 0 0 1 1 |  |  |
|  | **Model 9** |  |  |  |
| 4 | Housing stability | 2 3 3 1 | -34806.05 | -34711.92 |
|  | Quality of life | 3 3 3 3 |  |  |
|  | Community functioning ability | 2 3 3 3 |  |  |
|  | Substance use severity | 0 0 1 1 |  |  |
|  | **Model 10** |  |  |  |
| 4 | Housing stability | 3 3 3 1 | -34863.22 | -34769.10 |
|  | Quality of life | 2 3 3 3 |  |  |
|  | Community functioning ability | 2 3 3 3 |  |  |
|  | Substance use severity | 0 0 1 1 |  |  |
|  | **Model 11** |  |  |  |
| 4 | Housing stability | 1 3 3 2 | -34782.63 | -34690.22 |
|  | Quality of life | 2 3 3 3 |  |  |
|  | Community functioning ability | 2 3 3 3 |  |  |
|  | Substance use severity | 0 0 1 1 |  |  |
| a. Model selected | | | | |

**Supplementary Table 3. Parameters of good classification and accuracy for the assessed well-being multi-trajectory models.**

|  |  |  | **Parameters of good classification and accuracy for the multi-trajectory models** | | | |
| --- | --- | --- | --- | --- | --- | --- |
| **Multi-trajectory** | **Model growth shape** | **Multi-trajectory groups**  **within the model** | **n** | **Group Average Posterior Probability** | **Weighted (posterior probability) Odds of correct classification** | **Total group Probability** |
| **Model 1** |  |  |  |  |  |  |
| Housing stability | 2 3 3 1 | 1 | 127 | 0.94 | 46.71 | 0.240 |
| Quality of life | 2 3 3 3 | 2 | 209 | 0.90 | 14.67 | 0.379 |
| Community functioning ability | 2 3 3 3 | 3 | 109 | 0.88 | 29.90 | 0.197 |
| Substance use severity | 0 0 1 1 | 4 | 98 | 0.91 | 45.29 | 0.185 |
| **Model 2** |  |  |  |  |  |  |
| Housing stability | 1 3 3 2 | 1 | 115 | 0.91 | 35.96 | 0.212 |
| Quality of life | 3 3 3 3 | 2 | 188 | 0.90 | 16.46 | 0.348 |
| Community functioning ability | 3 3 3 3 | 3 | 97 | 0.89 | 37.73 | 0.177 |
| Substance use severity | 1 0 0 1 | 4 | 143 | 0.92 | 33.40 | 0.264 |
| **Model 3** |  |  |  |  |  |  |
| Housing stability | 3 3 3 1 | 1 | 142 | 0.93 | 38.25 | 0.266 |
| Quality of life | 2 3 3 2 | 2 | 118 | 0.91 | 35.96 | 0.220 |
| Community functioning ability | 2 2 3 3 | 3 | 144 | 0.94 | 41.02 | 0.269 |
| Substance use severity | 1 1 1 0 | 4 | 139 | 0.88 | 21.63 | 0.245 |
| **Model 4** |  |  |  |  |  |  |
| Housing stability | 2 3 3 1 | 1 | 138 | 0.92 | 34.10 | 0.255 |
| Quality of life | 2 3 3 3 | 2 | 127 | 0.91 | 33.75 | 0.239 |
| Community functioning ability | 2 3 3 3 | 3 | 138 | 0.94 | 47.61 | 0.259 |
| Substance use severity | 1 1 1 0 | 4 | 140 | 0.88 | 22.26 | 0.247 |
| **Model 5** |  |  |  |  |  |  |
| Housing stability | 2 3 3 1 | 1 | 127 | 0.94 | 48.24 | 0.243 |
| Quality of life | 2 3 3 3 | 2 | 210 | 0.90 | 15.12 | 0.382 |
| Community functioning ability | 2 3 3 3 | 3 | 106 | 0.87 | 29.52 | 0.188 |
| Substance use severity | 1 0 0 1 | 4 | 100 | 0.91 | 42.66 | 0.188 |
| **Model 6** |  |  |  |  |  |  |
| Housing stability | 2 3 3 1 | 1 | 133 | 0.92 | 37.61 | 0.246 |
| Quality of life | 2 3 3 3 | 2 | 120 | 0.92 | 38.32 | 0.224 |
| Community functioning ability | 2 2 3 3 | 3 | 139 | 0.94 | 46.07 | 0.262 |
| Substance use severity | 0 0 1 1 | 4 | 151 | 0.89 | 22.08 | 0.267 |
| **Model 7^a^** |  |  |  |  |  |  |
| Housing stability | **2 3 3 1** | **1** | **136** | **0.92** | **36.16** | **0.252** |
| Quality of life | **2 3 3 3** | **2** | **123** | **0.92** | **36.77** | **0.231** |
| Community functioning ability | **3 3 3 3** | **3** | **136** | **0.94** | **48.32** | **0.256** |
| Substance use severity | **0 0 1 1** | **4** | **148** | **0.89** | **21.92** | **0.261** |
| **Model 8** |  |  |  |  |  |  |
| Housing stability | 2 3 3 1 | 1 | 130 | 0.94 | 45.33 | 0.246 |
| Quality of life | 2 2 3 3 | 2 | 216 | 0.90 | 13.72 | 0.386 |
| Community functioning ability | 2 3 3 3 | 3 | 110 | 0.88 | 30.13 | 0.200 |
| Substance use severity | 0 0 1 1 | 4 | 87 | 0.92 | 56.35 | 0.168 |
| **Model 9** |  |  |  |  |  |  |
| Housing stability | 2 3 3 1 | 1 | 123 | 0.91 | 36.42 | 0.228 |
| Quality of life | 3 3 3 3 | 2 | 185 | 0.89 | 16.27 | 0.338 |
| Community functioning ability | 2 3 3 3 | 3 | 146 | 0.92 | 32.17 | 0.267 |
| Substance use severity | 0 0 1 1 | 4 | 89 | 0.91 | 47.23 | 0.168 |
| **Model 10** |  |  |  |  |  |  |
| Housing stability | 3 3 3 1 | 1 | 132 | 0.93 | 41.17 | 0.247 |
| Quality of life | 2 3 3 3 | 2 | 211 | 0.90 | 14.17 | 0.383 |
| Community functioning ability | 2 3 3 3 | 3 | 107 | 0.89 | 32.01 | 0.194 |
| Substance use severity | 0 0 1 1 | 4 | 93 | 0.91 | 47.89 | 0.176 |
| **Model 11** |  |  |  |  |  |  |
| Housing stability | 1 3 3 2 | 1 | 120 | 0.92 | 39.80 | 0.222 |
| Quality of life | 2 3 3 3 | 2 | 134 | 0.90 | 28.87 | 0.242 |
| Community functioning ability | 2 3 3 3 | 3 | 132 | 0.95 | 55.38 | 0.252 |
| Substance use severity | 0 0 1 1 | 4 | 157 | 0.90 | 22.27 | 0.284 |
| a. Model selected | | | | | | |

**Supplementary Table 4. Model growth parameters estimates for the selected well-being multi-trajectory group model, AH/CS study participants, Toronto site.**

|  | **Overall multi-trajectories model** | | | | |
| --- | --- | --- | --- | --- | --- |
| **Measure 1: Housing Stability** | **Housing stability: Logistic model** | | | | |
|  | **Model Growth Parameters (Standard Errors)** | | | | |
| **Trajectory groups** | Intercept (0)  Estimate (SE) | Linear (1)  Estimate (SE) | Quadratic (2)  Estimate (SE) | | Cubic (3)  Estimate (SE) |
| 1 | -2.22(0.20) | 0.11(0.01) | 0.00(0.00) | | ---------- |
| 2 | -3.21(0.36) | 0.54(0.06) | -0.02(0.00) | | 0.00(0.00) |
| 3 | -3.72(0.39) | 0.62(0.07) | -0.02(0.00) | | 0.00(0.00) |
| 4 | -2.04(0.16) | 0.05(0.00) | ---------- | | ---------- |
| **Measure 2:**  **Quality of life** | **Quality of life: Censored Normal model** | | | | |
|  | **Model Growth Parameters (Standard Errors)** | | | | |
| **Trajectory groups** | Intercept (0)  Estimate (SE) | Linear (1)  Estimate (SE) | | Quadratic (2)  Estimate (SE) | Cubic (3)  Estimate (SE) |
| 1 | 64.20(1.46) | 0.66(0.11) | | -0.01(0.00) | ---------- |
| 2 | 61.73(1.67) | 1.36(0.23) | | -0.04(0.01) | 0.00(0.00) |
| 3 | 83.07(1.57) | 2.08(0.21) | | -0.06(0.01) | 0.00(0.00) |
| 4 | 85.62(1.74) | 1.19(0.23) | | -0.03(0.01) | 0.00(0.00) |
|  |  | *Sigma (SE)* | | *p-value* |  |
|  |  | 18.14(0.22) | | 0.0000 |  |
| **Measure 3:**  **Community functioning** | **Community functioning ability: Censored Normal model** | | | | |
|  | **Model Growth Parameters (Standard Errors)** | | | | |
| **Trajectory groups** | Intercept (0)  Estimate (SE) | Linear (1)  Estimate (SE) | | Quadratic (2)  Estimate (SE) | Cubic (3)  Estimate (SE) |
| 1 | 52.45(0.50) | 0.27(0.07) | | -0.01(0.00) | 0.00(0.00) |
| 2 | 55.42(0.53) | 0.47(0.07) | | -0.02(0.00) | 0.00(0.00) |
| 3 | 57.04(0.49) | 0.78(0.07) | | -0.03(0.00) | 0.00(0.00) |
| 4 | 53.11(0.51) | 0.44(0.07) | | -0.02(0.00) | 0.00(0.00) |
|  |  | *Sigma (SE)* | | *p-value* |  |
|  |  | 5.97(0.07) | | 0.0000 |  |
| **Measure 4:**  **Substance use severity** | **Substances use severity: Logistic model** | | | | |
|  | **Model Growth Parameters (Standard Errors)** | | | | |
| **Trajectory groups** | Intercept (0)  Estimate (SE) | Linear (1)  Estimate (SE) | | Quadratic (2)  Estimate (SE) | Cubic (3)  Estimate (SE) |
| 1 | 0.08(0.08) | ---------- | | ---------- | ---------- |
| 2 | -1.76(0.16) | ---------- | | ---------- | ---------- |
| 3 | -1.55(0.16) | -0.03(0.01) | | ---------- | ---------- |
| 4 | -1.29(0.18) | -0.02(0.01) | |  |  |

**Supplementary Table 5. Variables with missing imputed values**

| **Variable** | **Number of observations with Complete values** | **Number of observations with Incomplete values** | **Number of observations with imputed values** | **Total**  **(Completed data)** |
| --- | --- | --- | --- | --- |
| ACES score | 444 | 99 | 99 | 543 |
| Resilience score (CD_RISC2 scale) | 470 | 73 | 73 | 543 |
| Mental health Symptoms severity (CSI score) | 523 | 20 | 20 | 543 |
| Education level | 524 | 19 | 19 | 543 |
| Lifetime of homelessness | 513 | 30 | 30 | 543 |
| History of brain trauma | 520 | 23 | 23 | 543 |
| History of discrimination experiences within the health care settings | 523 | 20 | 20 | 543 |

**Supplementary Table 6. Test for equal distribution across observed, imputed and completed values for numeric variables in the last five imputed datasets (m).**

| **Imputed variable** | Ksmirnov (test for equality of distribution functions): p-value in combined K-S |
| --- | --- |
| **ACE score** |  |
| *m=96* | 0.890 |
| *m=97* | 0.959 |
| *m=98* | 0.941 |
| *m=99* | 0.803 |
| *m=100* | 0.718 |
| **Resilience score** |  |
| *m=96* | 0.987 |
| *m=97* | 0.857 |
| *m=98* | 1.000 |
| *m=99* | 0.994 |
| *m=100* | 0.491 |
| **CSI score** |  |
| *m=96* | 0.973 |
| *m=97* | 0.820 |
| *m=98* | 0.698 |
| *m=99* | 0.659 |
| *m=100* | 0.913 |

**Supplementary Table 7. Comparison of the proportion in the observed, imputed and completed dataset for the years of lifetime homelessness in the last five imputed datasets.**

| **Proportion of the selected imputed variable** | | | | |
| --- | --- | --- | --- | --- |
| **Last 5 imputed datasets** | **Lifetime homelessness** | **Observed** | **Imputed** | **Completed** |
| *m=96* | <3 years | 0.456 | 0.200 | 0.442 |
|  | ≥3 years | 0.544 | 0.800 | 0.558 |
| *m=97* | <3 years | 0.456 | 0.267 | 0.446 |
|  | ≥3 years | 0.544 | 0.733 | 0.554 |
| *m=98* | <3 years | 0.456 | 0.467 | 0.457 |
|  | ≥3 years | 0.544 | 0.533 | 0.543 |
| *m=99* | <3 years | 0.456 | 0.300 | 0.448 |
|  | ≥3 years | 0.544 | 0.700 | 0.552 |
| *m=100* | <3 years | 0.456 | 0.400 | 0.453 |
|  | ≥3 years | 0.544 | 0.600 | 0.547 |

**Supplementary Table 8. Comparison of the proportion in the observed, imputed and completed dataset for the years of education in the last five imputed datasets.**

|  | **Proportion of the selected imputed variable** | | | |
| --- | --- | --- | --- | --- |
| **Last 10 Imputed datasets** | **Education level** | **Observed** | **Imputed** | **Completed** |
| *m=96* | Attended some middle/high school | 0.483 | 0.421 | 0.481 |
|  | Completed high school | 0.187 | 0.158 | 0.186 |
|  | Attended/completed graduate/postgraduate | 0.330 | 0.421 | 0.333 |
| *m=97* | Attended some middle/high school | 0.483 | 0.579 | 0.486 |
|  | Completed high school | 0.187 | 0.053 | 0.182 |
|  | Attended/completed graduate/postgraduate | 0.330 | 0.368 | 0.331 |
| *m=98* | Attended some middle/high school | 0.483 | 0.421 | 0.481 |
|  | Completed high school | 0.187 | 0.158 | 0.186 |
|  | Attended/completed graduate/postgraduate | 0.330 | 0.421 | 0.333 |
| *m=99* | Attended some middle/high school | 0.483 | 0.316 | 0.477 |
|  | Completed high school | 0.187 | 0.368 | 0.193 |
|  | Attended/completed graduate/postgraduate | 0.330 | 0.316 | 0.330 |
| *m=100* | Attended some middle/high school | 0.483 | 0.526 | 0.484 |
|  | Completed high school | 0.187 | 0.158 | 0.186 |
|  | Attended/completed graduate/postgraduate | 0.330 | 0.316 | 0.330 |

**Supplementary Table 9. Comparison of the proportion in the observed, imputed and completed dataset for the history of brain trauma in the last five imputed datasets.**

| **Proportion of the selected imputed variable** | | | | |
| --- | --- | --- | --- | --- |
| **Last five Imputed datasets** | **History of brain trauma** | **Observed** | **Imputed** | **Completed** |
| *m=96* | No | 0.460 | 0.565 | 0.464 |
|  | Yes | 0.540 | 0.435 | 0.536 |
| *m=97* | No | 0.460 | 0.391 | 0.457 |
|  | Yes | 0.540 | 0.609 | 0.543 |
| *m=98* | No | 0.460 | 0.391 | 0.457 |
|  | Yes | 0.540 | 0.609 | 0.543 |
| *m=99* | No | 0.460 | 0.348 | 0.455 |
|  | Yes | 0.540 | 0.652 | 0.545 |
| *m=100* | No | 0.460 | 0.391 | 0.457 |
|  | Yes | 0.540 | 0.609 | 0.543 |

**supplementary Table 10. Comparison of the proportion in the observed, imputed and completed dataset for the history of discrimination in healthcare settings in the last five imputed datasets.**

| **Proportion of the selected imputed variable** | | | | |
| --- | --- | --- | --- | --- |
| **Last five Imputed datasets** | **History of discrimination experienced in healthcare settings** | **Observed** | **Imputed** | **Completed** |
| *m=96* | No | 0.604 | 0.550 | 0.602 |
|  | Yes | 0.396 | 0.450 | 0.398 |
| *m=97* | No | 0.604 | 0.550 | 0.602 |
|  | Yes | 0.396 | 0.450 | 0.398 |
| *m=98* | No | 0.396 | 0.350 | 0.394 |
|  | Yes | 0.396 | 0.350 | 0.394 |
| *m=99* | No | 0.604 | 0.750 | 0.610 |
|  | Yes | 0.396 | 0.250 | 0.390 |
| *m=100* | No | 0.604 | 0.750 | 0.610 |
|  | Yes | 0.396 | 0.250 | 0.390 |
